# Evidence for neuroprotective properties of human umbilical cord blood cells after neuronal hypoxia *in vitro*

**DOI:** 10.1186/1471-2202-9-30

**Published:** 2008-02-29

**Authors:** Susann Hau, Doreen M Reich, Markus Scholz, Wilfried Naumann, Frank Emmrich, Manja Kamprad, Johannes Boltze

**Affiliations:** 1 Fraunhofer-Institute for Cell Therapy and Immunology, Deutscher Platz 5e, 04103 Leipzig, Germany; 2 University of Leipzig, Institute of Clinical Immunology and Transfusion Medicine, Johannisallee 30, 04103 Leipzig, Germany; 3 University of Leipzig, Faculty of Biology, Pharmacy and Psychology, Institute of Zoology II, Talstrasse 33, 04103 Leipzig, Germany; 4 University of Leipzig, Institute of Medical Informatics, Statistics and Epidemiology, Haertelstrasse 16-18, 04107 Leipzig, Germany; 5 Translational Centre for Regenerative Medicine, Philipp-Rosenthal-Strasse 55, 04103 Leipzig, Germany

## Abstract

**Background:**

One of the most promising options for treatment of stroke using adult stem cells are human umbilical cord blood (HUCB) cells that were already approved for therapeutic efficacy *in vivo*. However, complexity of animal models has thus far limited the understanding of beneficial cellular mechanisms. To address the influence of HUCB cells on neuronal tissue after stroke we established and employed a human *in vitro *model of neuronal hypoxia using fully differentiated vulnerable SH-SY5Y cells. These cells were incubated under an oxygen-reduced atmosphere (O_2_< 1%) for 48 hours. Subsequently, HUCB mononuclear cells (MNC) were added to post-hypoxic neuronal cultures. These cultures were characterized regarding to the development of apoptosis and necrosis over three days. Based on this we investigated the therapeutic influence of HUCB MNC on the progression of apoptotic cell death. The impact of HUCB cells and hypoxia on secretion of neuroprotective and inflammatory cytokines, chemokines and expression of adhesion molecules was proved.

**Results:**

Hypoxic cultivation of neurons initially induced a rate of 26% ± 13% of apoptosis. Hypoxia also caused an enhanced expression of Caspase-3 and cleaved poly(ADP-ribose) polymerase (PARP). Necrosis was only detected in low amounts. Within the next three days rate of apoptosis in untreated hypoxic cultures cumulated to 85% ± 11% (p ≤ 0.001). Specific cytokine (VEGF) patterns also suggest anti-apoptotic strategies of neuronal cells. Remarkably, the administration of MNC showed a noticeable reduction of apoptosis rates to levels of normoxic control cultures (7% ± 3%; p ≤ 0.001). In parallel, clustering of administered MNC next to axons and somata of neuronal cells was observed. Furthermore, MNC caused a pronounced increase of chemokines (CCL5; CCL3 and CXCL10).

**Conclusion:**

We established an *in vitro *model of neuronal hypoxia that affords the possibility to investigate both, apoptotic neuronal cell death and neuroprotective therapies. Here we employed the therapeutic model to study neuroprotective properties of HUCB cells.

We hypothesize that the neuroprotective effect of MNC was due to anti-apoptotic mechanisms related to direct cell-cell contacts with injured neuronal cells and distinct changes in neuroprotective, inflammatory cytokines as well as to the upregulation of chemokines within the co-cultures.

## Background

Acute ischemic stroke is characterised by the immediate depletion of oxygen and glucose in brain tissue. A residual cerebral blood flow (CBF) of ≤ 6 cm^3 ^× 100 g^-1 ^× min^-1 ^representing severe ischemia is associated with a nearly total loss of energy on vulnerable neurons. Ischemia therefore rapidly culminates in the formation of a necrotic core [1]. In the penumbra, mild ischemia (CBF 11–20 cm^3 ^× 100 g^-1 ^× min^-1^) leads to the activation of complex neurochemical cascades of cell death, mainly apoptosis. In principle these apoptotic cascades are reversible and form an important aspect of the penumbra concept, which is the major target of therapeutic interventions [2,3]. Recent findings indicate that transplantation of external cell fractions could accompany established therapeutic procedures limited by narrow time windows [4], but the underlying processes are still rather unclear.

Our insights into pathophysiological processes and new therapeutic strategies have mostly been obtained from animal models of focal cerebral ischemia [5,6] and rodent organotypic hippocampal slice cultures [7-9]. However, the complexity of those systems has limited the detailed understanding of mechanisms related to ischemic brain injury [10] and possible interfering effects of cellular therapies [11] so far. Furthermore, results obtained from rodent models are not completely and unobjectionably transferable to human therapy [12,13]. Consequently, experimental expenditure and ethical considerations demand *in vitro *models representing the main properties of stroke-related processes as neuronal apoptosis to accompany more complex model systems. This would allow to answer explicit questions concerning the role of cell-cell interactions and production of metabolites to verify observations made in *in vivo *models. It furthermore offers the possibility to precisely manipulate extra cellular environments.

Well described human neuronal cell lines exhibit a multitude of characteristics of typical central-nervous-system (CNS) neurons, overall cell material can be achieved in large quantities. Therefore, human neuronal cell lines, such as the teratocarcinoma NT-2 cell line, became useful tools to study the effects of hypoxic conditions on neurons [14]. However, the utilisation of NT-2 neuronal cultures is restricted by time-consuming and expensive differentiation periods of up to 44–54 days [15,16] that are also sensitive to environmental disturbances. In contrast, the SH-SY5Y neuroblastoma cell line was shown to be differentiated into neuronal cells within a comparatively short time of 16 days [17]. Furthermore, the cell line fits major relevant criteria (high vulnerability, irreversible differentiation into pure neuronal cells) to serve as a model of hypoxic injury of central neurons [18]. Hence, our exclusive human model of neuronal hypoxia forms the basis to identify possible anti-apoptotic neuroprotective potentials of therapeutic supplements. Mononuclear cells (MNC) from human umbilical cord blood (HUCB) were shown to improve functional outcome of animals after focal cerebral ischemia. The cellular effects causing the observed benefits are not fully understood for these cells [19,20]. In this context we investigated whether injured post-hypoxic neuronal cells or MNC initiated an anti-apoptotic response mediated by cytokines or chemokines.

## Results

### After differentiation SH-SY5Y exhibited neuronal morphology and specific neuronal markers

**Figure 1:**
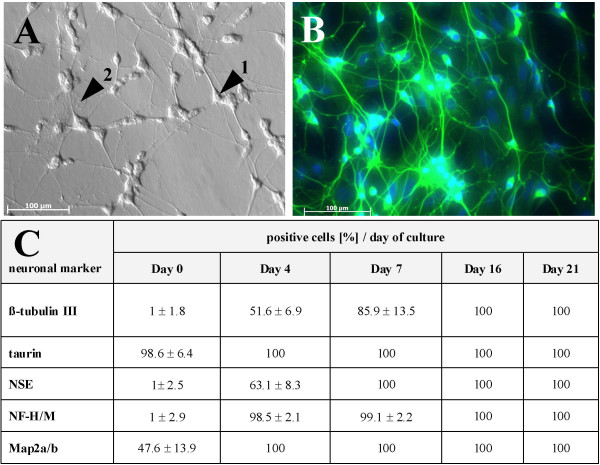
**Phase contrast and fluorescence imaging of fully differentiated SH-SY5Y cells and the development of neuronal markers**. Phase contrast image of fully matured SH-SY5Y cells at Day 16 (A) after seeding. Phase-bright neuronal cells (arrow 1) with long dendrites forming a dense neuronal network and cells with large cytoplasm and no axonal extensions (arrow 2). Immunocytofluorescence micrograph of fully differentiated SH-SY5Y cells (Day 16) shows cultures stained positive for the neuronal marker β-tubulin III (FITC, green) and nuclei (DAPI, blue) represented in B. Time course of detection of neuronal markers (β-tubulin III, taurin, NSE, NF-H/M and Map2a/b) on SH-SY5Y cells during the differentiation period at Day 0, 4, 7, 16 and 21 (C).

Sixteen days of differentiation yielded cultures of fully matured neuronal cells shown in Fig. 1A, B. Following the seventh day of differentiation, the majority of SH-SY5Y cells were stained positive for the specific neuronal markers (β-tubulin III, taurin I, neuron-specific enolase [NSE], neurofilament [NF] H/M and microtubule-associated proteins [Map] 2a/b). At Day 16 all cells exhibited all of these markers. Time course of marker expression showing continuously increasing stages of differentiation is given in Fig. 1C. Markers showed typical localisation to cytoplasm and dendrites. The explicit majority of differentiated SH-SY5Y cells (73% ± 11%) resembled typical neuronal morphology with round phase-bright somata and long, terminal- branched dendrites forming a dense network (Fig. 1A, arrow 1). However, the neuronal culture is also characterised by a cell type (only 27% ± 11%) which shows abundant cytoplasm and a lack of axonal extensions (Fig. 1A, arrow 2). Remarkably, no differences were noted with regard to expression of neuronal markers. Furthermore, the number of this type of cells remained constant during the culture time.

### Absence of proliferation after termination of differentiation

**Figure 2:**
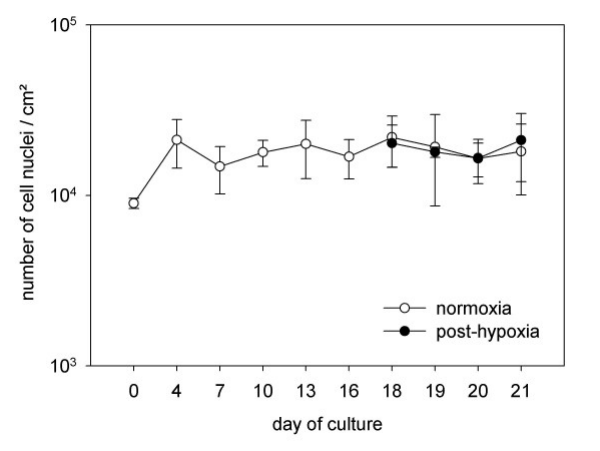
**Progression of the number of nuclei after differentiation period and effect of hypoxia on cell count**. Nuclei were stained with DAPI and counted in fluorescence micrographs. Data were calculated from three independent experiments including multiple wells, each based on 25 micrographs. The white circles represent cultures that were cultivated under continuously normoxic conditions. The black circles illustrate the progression of the number of nuclei in post-hypoxic cultures. Under both conditions number of nuclei remained stable.

At Day 4 of differentiation the total number of SH-SY5Y cells, counted by nuclear staining with DAPI, increased by twofold (21.2 ± 6.7 × 10^3^/cm^2^) since seeding. Beginning on the seventh day of culture numbers of nuclei remained nearly stable (18.2 ± 2.2 × 10^3^/cm^2^). Subsequent to hypoxia, there was no significant alteration in numbers of counted nuclei. Hence, the influence of oxygen deprivation induced no further proliferation and also no significant loss of cells in comparison to normoxic control cultures (Fig. 2). Therefore all following results should be seen in the context of the nearly constant numbers of neuronal cells under normoxic and hypoxic cultivation conditions. It is of note that the amount of stained nuclei as the equivalent of adherent cells gives no information about the physiologic status of these cells. On the whole the cultures include cells in a viable, apoptotic or necrotic state.

### Hypoxia induced apoptosis in the majority of neuronal cells

**Figure 3:**
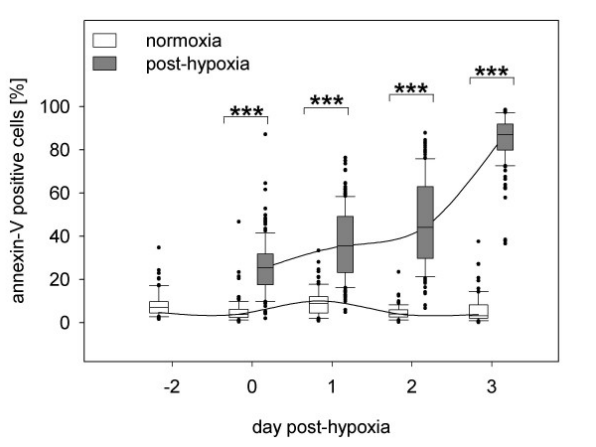
**Temporal progression of annexin-V positive cells in post-hypoxic and normoxic cultures**. Results are derived from three independent experiments. For each experiment 75 pictures were taken. Consistently, annexin-V positive (apoptotic) values in post-hypoxic cultures are markedly higher compared to control cultures increasing to an average level of 85% ± 11% within three days. The number of apoptotic cells did not vary significantly in normoxic cultures.

**Figure 4:**
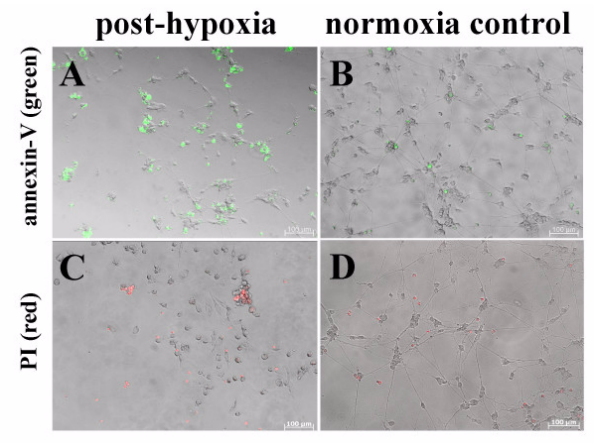
**Long term effect of 48 hours of hypoxia on apoptosis (A-B) and necrosis (C-D)**. Cultures at Day 3 after hypoxia were compared with normoxic cultures of the same age. Combined phase contrast/fluorescence micrographs of neuronal post-hypoxic cultures (A) and normoxic control cultures (B) show a conspicuous increase of apoptotic cells (annexin-V-staining, green fluorescence) and morphologic changes due to hypoxia (cell debris, retraction of dendrites). Propidium iodide (PI, red fluorescence) staining shows the influence of hypoxia (C) on the number of necrotic cells and cells in a late state of apoptosis compared to control cultures (D). In contrast to the level of apoptosis, there was no clear difference in the number necrotic cells following both culture conditions.

Post-hypoxic and normoxic control cultures exhibited pronounced differences in the quantity of apoptotic cells as well as in cell morphology. Hypoxic conditions for 48 hours induced an initial apoptosis rate of 26% ± 13%. There was a continuous increase in the apoptotic cell fraction to 85% ± 11% within 3 days post-hypoxia as compared to control cultures (p ≤ 0.001; Fig. 3). The clearest effects of oxygen deficiency were seen three days following induction of hypoxia as shown by annexin-V staining (green fluorescence, Fig. 4A and 4B). Additionally, post-hypoxic cultures were characterised by retracted dendrites, indicating a loss of multiple cell-cell contacts. Debris and apoptotic bodies were found in most culture dish areas, evidencing late stage of apoptosis (Fig. 4A). In contrast, normoxic cultures displayed a much more reduced amount of apoptotic cells (Fig. 4B). Over the whole time, apoptosis in these control cultures remained stable at about 7% ± 3% (Fig. 3).

**Figure 5:**
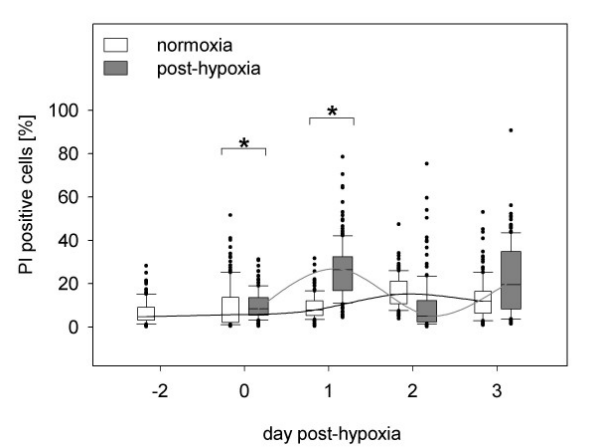
**The progression of PI positive cells in post-hypoxic and normoxic SH-SY5Y cultures**. Results are derived from three independent experiments, in which 75 pictures were taken for each analysis. Significant increases in the rate of PI positive cells were observed only at Day 0 (10% ± 6%) and Day 1 (27% ± 13%) compared to normoxic cultures (Day 0: 9% ± 10%); Day 1: 9% ± 6%). Note PI stains necrotic and late apoptotic cells. No significant difference of necrosis rate was detected of post-hypoxic cultures as well as in control cultures during the observation period.

After hypoxia, on Day 0 and Day 1, number of late apoptotic/necrotic cells significantly increased up to 27% ± 13%. Further progression of propidium iodide (PI) positive cells in post-hypoxic cultures resulted in 23 ± 16% on Day 3 (Fig. 5). By comparison, in normoxic control cultures, necrosis levels remained stable below 17% and therefore did not statistically differ from post-hypoxic cultures on this time point. This fact was corroborated by images that show no observable deviation in the amount of necrotic and late apoptotic cells, as indicated by PI staining (red fluorescence, Fig. 4C and 4D).

In general, an increased release of calcein-AM also indicated a strong decrease of neuronal cell viability which verifies the results of the apoptosis and necrosis rates (data not shown).

### Hypoxia increased quantities of Caspase-3 and cleaved PARP immediately and secretion of VEGF in delay

**Figure 6:**
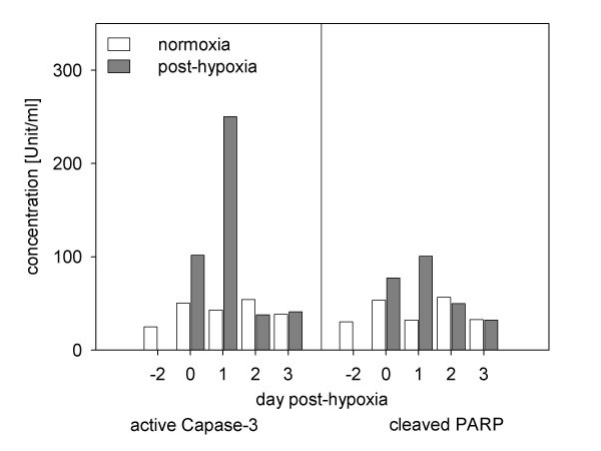
**Effect of 48 hours of hypoxia on the concentration of active Caspase-3 and cleaved PARP**. Data are taken in a time course of three days after hypoxia and are based on pooled lysates of the totality of SH-SY5Y cells taken out of 12 individual wells. Data are expressed in units of protein [active Caspase-3] and cleaved protein [PARP] per millilitres. Hypoxia induced apoptosis specific proteins (Caspase-3, cleaved PARP) in a time-related manner.

**Figure 7:**
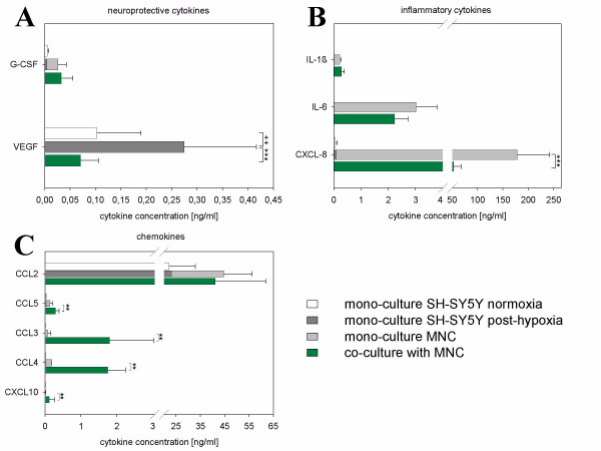
**Cytokine profile in hypoxic injured SH-SY5Y cells and in direct co-cultures with MNC at Day 3**. Cytokine concentrations were measured in supernantes via cytometric bead array. Data originate from five independent experiments and are expressed as ng/mg. "+" indicates significant differences in cytokine concentration of post-hypoxic SH-SY5Y mono-cultures compared to normoxic controls. Double bars are the sum of cytokine concentration from mono-cultures. "*" shows significant differences in cytokine concentration in co-cultures with MNC compared to the total of mono-cultures. Note different scaling.

Bar charts in Figure 6 show the concentrations of Caspase-3 and cleaved poly(ADP-ribose) polymerase (PARP). In post-hypoxic cultures apoptotic proteins drastically rose after oxygen deprivation. The highest concentration of Caspase-3 was measured directly after hypoxia on Days 0 and 1 (2-fold and 5.8-fold, respectively). From Day 2 on, the level of active Caspase-3 sharply decreased. The distribution of cleaved PARP levels showed patterns similar to Caspase-3. This decline was accompanied by a secretion of VEGF on Day 3 after hypoxia (Fig. 7).

### Adhesion molecules (L1, NCAM and ICAM-1) were upregulated after hypoxia

**Figure 8:**
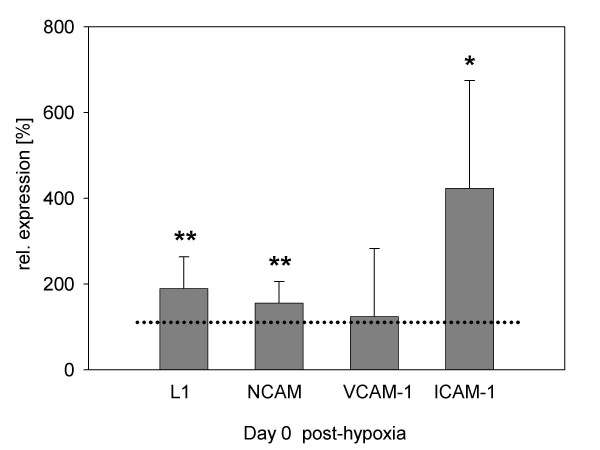
**Expression of adhesion molecules after 48 hours of hypoxic cultivation**. Fluorescence intensities of cell lysates are shown after subtraction of specific isotype values with respect to normoxia. Data arise from six independent experiments. The dotted line indicates level of adhesion molecule expression (100%) in normoxic control cultures. Note statistically significant upregulation of L1, NCAM and ICAM-1 post-hypoxia.

Independent of cultivation conditions immunofluorescence analysis revealed that nearly all (98%) of the cells were positive for neurite cell adhesion molecule (L1; 98.3 ± 0.1%) and Neural Cell Adhesion Molecule (NCAM; 99 ± 0.8%) at Day 0. L1 and NCAM were detected on somata and multiple dendrites. Furthermore, a few of the neuronal cells also expressed Vascular Cell Adhesion Molecule (VCAM-1; 5.7%) and Intercellular Adhesion Molecule (ICAM-1; 14.6%) on their somata and dendrites. There was no change in relative numbers of marked cells and expression patterns in post-hypoxic cultures. To quantify the density of adhesion molecule expression, highly sensitive fluorescence measurement of supernatants of lysed cultures was performed. Here, comparative studies between cultures directly after 48 hours of hypoxia and normoxic control cultures showed a significant upregulation of L1, NCAM and ICAM-1 (Fig. 8). Hypoxic conditions determined a considerable increase in L1 and NCAM protein expressions of up to 189% ± 74% and 155% ± 50%, respectively. Hypoxia also strongly upregulated the ICAM-1 expression up to 424% ± 251%. The level of expression of VCAM-1 was not altered by the hypoxic environment. The continued degradation of the dendritic networks did not allow assaying the specific expression of adhesion molecules at late stages of post-hypoxic cultures.

### Co-culturing with HUCB MNC strongly reduced apoptosis in post-hypoxic neuronal cell cultures

**Figure 9:**
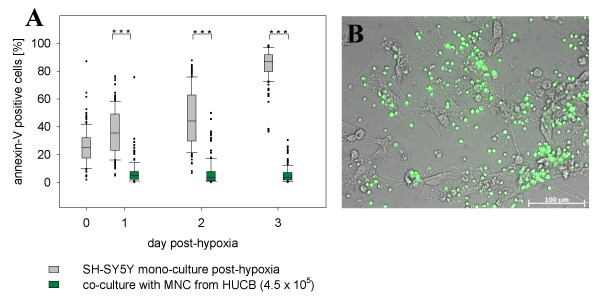
**Effect of co-culturing of MNC on ratio of neuronal apoptosis and preservation of neuronal networks**. Apoptosis was induced by 48 hours incubation of neuronal cells under hypoxic conditions. Afterwards 4.5 × 10^5 ^CFSE stained MNC were directly applied to neuronal cells (0.3 × 10^5^/well). For three days co-cultures were observed under normoxia. In co-cultures with MNC rate of apoptosis was clearly reduced compared to post-hypoxic cultures (A). Combined phase contrast and fluorescence micrograph of post-hypoxic neuronal cells and MNC (green) in direct co-culture (B).

We found noticeable neuroprotective properties of HUCB MNC in the co-culture experiments. Untreated post-hypoxic mono-cultures of neuronal cells showed 85% ± 11% of apoptosis after three days, whereas in co-cultures with MNC the rate of apoptosis was stable at a level of 6.3% ± 1% (p ≤ 0.001). This is comparable to normoxic control cultures (7% ± 3%; Fig. 9A). Photographs of co-cultures with MNC revealed clear effort of MNC to localise next to somata and branches of post-hypoxic neuronal cells. In the course of clustering MNC mostly avoided areas that were not settled by neuronal cells (Fig. 9B). Furthermore, the administration of MNC showed positive influence on the conservation of neuronal networks as compared to cultures that did not receive any cell therapy (Fig. 4A and 4C).

### MNC increased CCL5, CCL3, CCL4 and CXCL10 in post-hypoxic neuronal cultures

The investigation of neuroprotective cytokines (G-CSF, VEGF) (Fig. 7A), inflammatory cytokines (IL-1β, IL-6, CXCL8) (Fig. 7B) and chemokines (CCL2, CCL5, CCL3, CCL4, CXCL10) (Fig. 7C) in supernatants of normoxic neuronal cultures revealed considerable amounts of CXCL8, CCL2 and VEGF. Remarkably, after three days under hypoxic conditions only VEGF was strongly upregulated (about threefold increase). After administration of MNC, VEGF was downregulated regarding to the total concentration measured in mono-cultures of MNC and in post-hypoxic neuronal cells. Inflammatory cytokines as IL-1β, IL-6 and CXCL8 were produced by MNC. The secretion of cytokines such as IL-1β and IL-6 was not altered in post-hypoxic co-cultures, whereas CXCL8 was suppressed. Interestingly, the majority of chemokines was clearly upregulated in co-cultures with MNC. We could show that CCL5, CCL3, CCL4 and CXCL10 were increased up to the tenfold (CCL3, CCL4), whereas the concentration CCL2 was not regulated.

## Discussion

In this study, we introduce an experimental human in vitro model to investigate (i) the mechanisms of neuronal hypoxia and (ii) the interaction of neuronal cells with external stem cell-containing fractions as possible therapeutic tools. According to the differentiation protocol of Encinas et al. [17] we obtained fully matured neuronal cells after 16 days. Previous, orientating experiments showed typical response of differentiated SH-SY5Y cells to N-methyl-D-aspartate (NMDA; 300 μM) application and most pronounced vulnerability after 48 hours hypoxic incubation (data not shown). These post-hypoxic neuronal cultures can be employed already 18 days after the seeding of naive cells. Therefore the time period until the model of hypoxic neuronal cells is available is very short. In comparison to other approaches that provide assimilable hypoxic models using teratocarcinoma cell lines [16] the generation of our model is shortened by at least 10 days [21]. The proved expression of specific neuronal markers and the absence of any proliferation gave clear evidence of matured neuronal cells in the G0-phase of the cell cycle. This steady state allows making direct conclusions about the influence of any external manipulation because any change will most probably be due to these procedures. Hence, the model of neuronal hypoxia affords the possibility to investigate manifold questions concerning mechanisms triggered in response to hypoxia and therapeutic interventions e.g. application of HUCB MNC.

Following hypoxia, neuronal cultures displayed a correlation between morphological changes and increase of annexin-V positive cells as well as changes in adhesion molecule expression. The specific apoptosis marker annexin-V indicated that hypoxic cultivation preferentially induced apoptosis. Moreover, there was a continuous enlargement in number of apoptotic cells from initial 26% ± 13% until cultures almost completely consisted of apoptotic cells (85% ± 11%) within three days. Conforming to changes of morphology in post-hypoxic cultures, there was an enhanced release of calcein-AM also observable due to the loss of the integrity of membrane. This is typical for late stages of apoptosis as well as of necrosis. The model of neuronal hypoxia was approved by moderate rates of PI positive cells (23% ± 16% at Day 3 post-hypoxia). Therefore the overrun of 100% by the summation of annexin-V positive and PI positive cells is due to a proportion of cells in a late state of apoptosis that are positive for annexin-V and PI [22]. We focused on apoptosis because it is a reversible process which could be modulate by external stimulation [23,24]. Moreover after stroke cell death in the penumbra is predominantly considered to be apoptosis [25-28]. Therefore apoptosis is a therapeutic target of anti-apoptotic therapies like stem cells or cytokines [29-32].

Apoptosis in the penumbra takes place in a time span of about 72 hours after vessel occlusion in the rat after middle cerebral artery occlusion (MCAO) [33]. This time course is represented by the model system introduced in this study. The fact that there was no major alteration in the number in DAPI-positive nuclei underlines that the influence of hypoxia did not lead to an immediate destruction of the cells but allows investigating apoptosis and subsequent therapeutic interventions within 72 hours. These facts were also confirmed by characteristic changes in the morphology of post-hypoxic cells. There was a total retraction of dendrites and a resulting destruction of neuronal networks, grained cell surfaces and extended cell degradation. This damage was accompanied by an upregulation of neuronal adhesion molecules. We hypothesize that this upregulation indicates a cellular answer to compensate for a loss of direct cell-cell interaction as a consequence of the preceding hypoxic stress as described by several authors [34-36]. However, the final loss of intercellular networks could not be compensated by the increased expression of neuronal adhesion molecules alone.

Neuronal cell death after hypoxia is caused by membrane depolarisation subsequent to energy failure [37,38]. The resulting calcium overload is the initial point of the activation of manifold biochemical pathways that affect caspases, free radicals and gene expression [39,40]. The specific apoptotic proteins Caspase-3 and cleaved PARP were quantified to prove whether our microscopic observations are corroborated by biochemical pathways [41-43]. After 24 hours post-hypoxia, both marker proteins extensively increased. This was followed by a sharp drop in their expression level. This reduced amount of cleaved PARP might be due to an aggravating energy failure in the cell. This in turn is attributed to the high levels of cleaved PARP, evidenced above, which caused adenosintriphosphate (ATP) depletion [44,45] and which in this context prevents the enzymatic activity of caspases. Furthermore, an increase of the expression of VEGF, a key mediator of angiogenesis [46,47], was observed in higher concentrations in late post-hypoxic cultures. This might be a reflection of compensatory *in vivo *processes of revascularisation in our model [48,49]. VEGF is also known to be involved in neuronal protection through inhibition of Caspase-3 [50,51]. The time-delayed increased release of VEGF as an effect of hypoxia does not seem to influence the number of apoptotic cells in our cultures. This may be a result of concentrations of VEGF that did not reach efficient thresholds. In this context the measured reduction of caspases on Days 2 and 3 after hypoxia is caused by the already described ongoing energy failure in the post-hypoxic cells. However, the regulation of those apoptotic proteins is probably not related to VEGF mediated-effects.

The therapeutic benefit of cell administration after stroke has been demonstrated in numerous animal studies [52,53]. However, important questions in basic cellular and molecular mechanisms of neuronal cell death and its prevention after inadequate oxygen supply still remain unanswered. To understand the mechanisms of cellular neuroprotection after stroke we employed the model of neuronal hypoxia and applied MNC from HUCB.

*In vivo *MNC are supposed to differentiate into neuronal cells [54,55] to trophically support neuronal tissue through the production of growth factors [56], to support the new formation of synapses and migration as well as the differentiation of endogenous neuronal progenitors [54,55]. In our experiments, the administration of MNC from HUCB to post-hypoxic neuronal cultures showed remarkable beneficial effects. Over three days we could show a clear reduction of neuronal apoptosis, even to the level of normoxic control cultures (7% ± 3%). There are two observations that contribute to an explanation of neuroprotective mechanisms. First, MNC were preferentially located close to hypoxically injured neuronal cells. This phenomenon was facilitated through an intensive upregulation of ICAM-1 on neuronal cells which is the specific ligand for leukocyte cell adhesion molecule [LFA-1] expressed by all immune cell subsets [57]. However, the formation of cell chimera was not observed, as there were no CFSE-stained cells with neuronal morphology. In future experiments we will investigate whether blocking of adhesion molecules on the surface of neuronal cells can inhibit the co-localisation of MNC. Further it will be proved whether this influences the rate of apoptosis. An increase of apoptosis would be an evidence for the significance of direct cell-cell-contacts in context of therapeutic mechanisms of MNC. The second observation would be concomitance of an MNC indicated specific alteration in levels of soluble factors. That change might be held responsible for neuroprotection. Pronounced upregulation of chemokines (CCL5, CCL3, CCL4, CXCL10) might be causal for the enhanced effort of MNC to localise near neuronal structures. High concentrations of VEGF are known to be neuroprotective [58,59]. In contrast to post-hypoxic mono-cultures, we found no increase of VEGF after administration of MNC. It seems that presence of MNC inhibited the induction of elevated levels of VEGF and compensated its neuroprotective effects by other mechanisms. Cord blood contains distinct cell types capable to differentiate into neuronal cells [60]. However, the differentiation of stem cells (about 1% within the MNC fraction) was not expected because of the short time of co-cultivation that clearly undershoots time frames responsible for neuronal differentiation.

So far, it is unclear whether neuroprotection resulted from one of the observed effects or from a combination of spatial proximity and specific cytokine patterns. Consequently, further studies in indirect co-cultures will be necessary.

## Conclusion

Pathophysiological models developed from animal studies form the basis of our understanding of the development of stroke. *In vivo *data display a perfusion-related dependency of neuronal cell damage. Residual energy supply in the penumbra induces apoptosis, the early phases of which are reversible. Consequently, rescue of the penumbra is a major target of experimental stroke therapy. Data obtained from complex animal models of stroke strongly suggest that transplanted cells enhance neuronal survival.

We established a standardized human *in vitro *model of neuronal apoptotic cell death after hypoxia that can facilitate to address specific pathophysiological processes underlying hypoxic damage and cell-mediated neuroprotection more precisely.

Interestingly, transplanted MNC not only strongly decreased the ratio of apoptosis in neuronal cells but also triggered retaining neuronal characteristics such as forming networks. MNC clustered around post-hypoxic neuronal cells and induced an alteration in cytokine and chemokine concentrations. Our data suggest that the neuroprotective effects of MNC might result from direct cell-cell contacts and/or the adjustment of specific soluble mediators.

## Methods

### Cultivation and differentiation of neuronal cells

**Figure 10:**
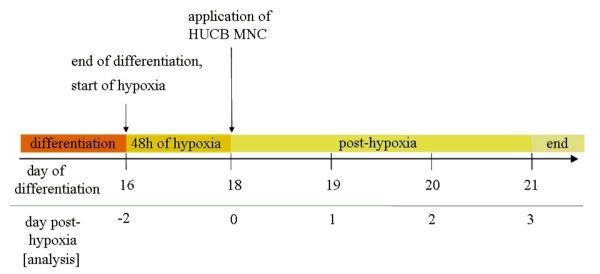
**Schematic illustration of experimental set up**. Sixteen days of differentiation were followed by 48 hours cultivation under hypoxic conditions. Following hypoxia cultures were maintained under normoxic conditions for three days and are referred as post-hypoxic. Subsequently, cultures were analysed daily.

All experiments were performed using SH-SY5Y human neuroblastoma cells (DSMZ, German Collection of Microorganisms and Cell Cultures, Braunschweig, Germany) between passages 4–7. The common medium was Dulbecco's Modified Eagle Medium (DMEM, high glucose 4.5 g/l, L-Glutamine 580 mg/l; PAA, Pasching, Austria) with penicillin G (10,000 U/ml; PAA Laboratories, Pasching, Austria) and streptomycin (10 mg/ml; PAA Laboratories, Pasching, Austria]. Cells were maintained in MM in a humidified atmosphere with 5.5% CO_2 _at 37°C (Table 1). When cultures achieved subconfluence, cells were subcultured with trypsin/EDTA (PAA Laboratories, Pasching, Austria). Differentiation was carried out according to the protocol of Encinas and colleagues [17] but was adapted as follows: cells were plated at an initial density of 0.9 × 10^4^/cm^2 ^in 16-mm-diameter cavity (Greiner Bio-One, Frickenhausen, Germany) and differentiated over a period of 16 days in relevant media and cultured thereafter according to Table 1. Cultivation procedure is illustrated by Fig. 10.

**Table 1:** Composition of culture media

			**Supplements in DMEM**				
			
**Day of culture**	**Culture condition**	**Description of media**	**FCS (15%)**	**RA (10 μM)**	**BDNF (5 ng/ml)**	**HSA (0.1%)**	**Ham's F12**
Seeding	Normoxia	Maintenance medium (MM)	x	-	-	-	-
1 – 4	Normoxia	Basic medium (BM)	x	x	-	-	-
5 – 16	Normoxia	Differentiation medium (DM)	-	x	x	x	x
16 – 18	Normoxia/hypoxia	Differentiation medium (DM)	-	x	x	x	x
18 – 21	Normoxia	Post-hypoxic medium (PHM)	-	-	x	x	x

The experimental design demanded varying culture media. Supplements in DMEM were purchased from: fetal calf serum (FCS, PAN-Biotech, Aidenbach, Germany), all-trans-retinoic acid (RA, Sigma-Aldrich, Steinheim, Germany), recombinant brain-derived neurotrophic factor (rhBDNF; ImmunoTools, Friesoythe, Germany), human serum albumin (HSA, PAN Biotech, Aidenbach, Germany).

### Immunocytofluorescence of neuronal markers

The primary antibodies used against neuronal epitopes were: β-tubulin III (rabbit 10 μg/ml; BD PharMingen, Heidelberg, Germany), taurin I and neurofilament (NF) H/M (rabbit 5 μg/ml and mouse 1:200; Chemicon, Hampshire, UK), neuron-specific enolase (NSE) and microtubule-associated proteins (Map) 2a/b (mouse 1:2 and mouse 5 μg/ml; Sigma-Aldrich, Munich, Germany). Fluorochrome-conjugated secondary antibodies were purchased from DAKO, Carpinteria, CA, USA (goat anti-mouse-PE [1:200] and pork anti-rabbit-FITC [1:30]). Indirect immunostaining was processed according to manufacturer's instructions.

### Hypoxia and post-hypoxic cultivation

Before enddifferentiated neuronal cells were exposed to hypoxia they were refreshed with DM. Hypoxic conditions were O_2 _< 1% (oxygen substituted with nitrogen) and lasted 48 hours in a 37°C tempered and humidified incubator (Binder GmbH, Tuttlingen). After hypoxia, cultures were supplied with PHM (Table 1) and were transferred back to normoxic conditions for the following three days (Fig. 10).

### Quantification of cell numbers by nuclear staining

The total number of cells was measured by nuclear staining with 4', 6-diamidino-2-phenylindole (DAPI, Invitrogen, Karlsruhe, Germany). Cells were washed and stained with 1 μg/ml (DAPI/Methanol) for 15 minutes at 37°C. 25 microphotographs of randomised fields were taken per cavity using a Zeiss fluorescence microscope (Carl Zeiss AG, Jena, Germany) equipped with Zeiss AxioVision-Software. The number of nuclei was automatically determined by means of Zeiss AutMess (Carl Zeiss AG, Jena, Germany).

### Cell viability assay

Apoptosis was determined via annexin-fluorescein isothiocyanate (FITC) or annexin-phycoerythrin (PE; both 1:20 reaction buffer; BD PharMingen, Heidelberg, Germany) using fluorescence microscopy. Necrosis was identified by propidium iodide (PI) staining (1 μg/ml phosphate-buffered saline [PBS]; Bender medSystems, Vienna, Austria). Both methods detect cells in a late state of apoptosis. Apoptosis and necrosis were ascertained in different cavities. Calcein-AM (40 μM/PBS; Invitrogen, Karlsruhe, Germany) was utilised for detection of living cells.

### Quantification of apoptotic proteins

BD Cytometric Bead Array for human apoptosis (Becton Dickinson, Erembodegem, Belgium) was used for the quantitative measurement of apoptotic proteins (cleaved PARP and Caspase-3). Manufacturer' s instructions were adapted to lyse cells directly within multi-well plates. For cell lyses differentiated cells were rinsed with PBS and incubated on ice in the provided buffer for 20 minutes.

### Determination of adhesion molecules by immunocytochemistry and cell-based fluorescence measurement

The cultures were stained with antibodies against CD56-phycoerythrin (PE; NCAM), CD171 (L1, both Becton Dickinson, Erembodegem, Belgium), CD54-PE (ICAM-1; Immunotech, Hamburg, Germany) and CD106-PE (VCAM-1; Southern Biotech, Birmingham, Alabama, USA) in order to investigate the distribution and localisation of adhesion molecules on differentiated neuronal cells. The staining protocol proceeded as follows: Medium (DM, Table 1) was removed and dishes were washed twice with PBS. The cells were incubated with primary labelled or unlabelled antibodies (1:100) for 10 minutes at 37°C and afterwards washed in PBS. Thereafter the cells were incubated with goat anti-mouse-PE (DAKO, Hamburg, Germany) antibody for 10 minutes at 37°C for CD171 detection. After labelling the cultures were immersed in PBS.

The spatial distribution of adhesion molecules was observed using fluorescence microscopy (Carl Zeiss AG, Jena, Germany). The density of molecule expression was measured in cell lysates. Therefore, antibody-labelled cultures were treated with 1% Triton × 100 (Ferak, Berlin, Germany)/PBS at 37°C. Whole-cell lysates were transferred to black 96-well-plates (Greiner Bio-One, Frickenhausen, Germany). PE-fluorescence signals were determined with a spectrafluorometer (Tecan Spectrafluor Plus, Tecan Trading AG, Switzerland) at an excitation wavelength of 488 nm and an emission wavelength of 590 nm. Specific isotypes and 1% Triton × 100 served as negative controls.

### Preparation of Human Umbilical Cord Blood (HUCB) samples

Cord blood samples were obtained anonymously in accordance to ethical prescripts immediately after delivery. HUCB samples of healthy full-term neonates were processed according experienced methods including density gradient separation using Lymphocyte Separation Medium (PAA Laboratories, Cölbe, Germany). Gained MNC fraction was stored by freezing in the gaseous phase of liquid nitrogen after the addition of FCS/8% dimethyl sulfoxide (Serumwerke Bernburg Inc., Bernburg, Germany). Prior to use cryopreserved MNC were thawed rapidly in 75 U/ml DNaseI/0.5 M MgCl_2 _(Roche Diagnostics GmbH, Mannheim, Germany/Sigma, Germany) and washed in RPMI (PAA Laboratories, Austria). Cell suspension was stained with carboxy fluoresceindiacetate succinimidyl ester (CFSE 5 μM; Molecular Probes, Inc., Eugene, OR, USA) for 10 minutes at 37°C.

### Co-culture of neuronal cells and MNC

Direct co-culturing of fully differentiated neuronal cells and MNC was carried out under normoxic conditions (37°C) over a period of 3 days following 48 hours of hypoxia. A total amount of 4.5 × 10^5 ^CFSE stained MNC were dissolved in 500 μl PHM and were added to the post-hypoxic neuronal cells (0.3 × 10^5^/cavity). The ratio of neuronal cells to MNC was 1:15.

### Cytokine profiling

The supernatants from normoxic cultures on Day 21 as well as from hypoxic cultures on Day 3 post-hypoxia were collected in order to characterise soluble factors produced by cultured cells. They were detected simultaneously by means of Becton Dickinson Cytometric Bead Array. Supernatants were tested for neuroprotective (Granulocyte Colony-Stimulating Factor [G-CSF], Vascular Endothelial Growth Factor [VEGF]) and inflammatory cytokines (Interleukin [IL]-1β, IL-6, CXCL8) as well as chemokines (CXCL10, CCL3, CCL4, CCL5, CCL2). The detection limit was 0.02 ng/ml, except for VEGF and CCL2 (0.04 ng/ml).

### Statistical analyses of data

Except for apoptosis and necrosis rates all results have been reported as mean values ± SD. Statistical differences were analysed by the Student s t-test or the Mann-Whitney rank sum-test. P values of ≤ 0.05 were considered statistically significant (* p ≤ 0.05, ** p ≤ 0.01, *** p ≤ 0.001). Apoptosis and necrosis rates were logit-transformed to obtain normally distributed quantities. The effects of time, experimental setting (hypoxia), experimental run and the investigated cavity were determined univariately, and, finally multivariately using a mixed-model approach with time and experimental setting as fixed effects and cavity and experimental run as random effects. Cytokine concentrations were compared between the normoxic and the hypoxic group by means of the Mann-Whitney rank sum test. Cytokine concentrations measured in co-cultures were compared with the sum of the concentrations obtained in the post-hypoxic neuronal cultures and in the MNC mono-cultures via a bootstrapping algorithm. This was performed by resampling and by the addition of concentrations of cytokines measured in post-hypoxic neuronal cultures and MNC mono- cultures. Results were compared with the Mann-Whitney rank sum test. The mean and standard deviations of the sum of the concentrations were also determined by bootstrapping. Bootstrapping analysis was performed using the statistical software package "R" [61,62]. Mixed-model analyses were performed using PROC MIXED from the statistical software package SAS 9.1 (SAS Institute Inc., Cary, NC, USA). Box plots (if applicable) and univariate analyses were determined using the software package SPSS (SPSS Inc., Chicago IL, USA).

## Authors' contributions

SH, DMR, MK and JB designed and coordinated the study. SH and DMR conducted all experimental work and wrote the manuscript. SH, DMR and MK analysed and interpreted the data. MS performed the statistical analysis. WN and FE critically revised this study. All authors read and approved the final version of the manuscript.
